# Role of Sentinel Node Surgery in Identifying Candidates for Adjuvant CDK4/6 Inhibitors: Implications of Choosing Wisely and ASCO Omission Criteria

**DOI:** 10.1245/s10434-026-20131-3

**Published:** 2026-07-08

**Authors:** Austin D. Williams, Walker Lyons, Lindsey B. Taylor, Cecilia Chang, Angela Jain, Melissa McShane, Allison A. Aggon, Alycia So, Jennifer Winn, Richard J. Bleicher

**Affiliations:** 1 Division of Breast Surgery, Department of Surgery, Fox Chase Cancer Center, Philadelphia, PA USA; 2 Department of Hematology/Oncology, Fox Chase Cancer Center, Philadelphia, PA USA; 3 Endeavor Health Research Institute, Evanston, IL USA; 4 Department of Surgery, Fox Chase Cancer Center, Philadelphia, PA USA

**Keywords:** Sentinel lymph node biopsy, Axillary surgery, Breast cancer, CDK4/6 inhibitors, Abemaciclib, Ribociclib, De-escalation, Adjuvant therapy

## Abstract

**Introduction:**

Recent trials have expanded eligibility for omission of sentinel lymph node biopsy (SLNB) in early-stage breast cancer, while adjuvant cyclin-dependent kinase 4/6 (CDK4/6) inhibitors improve outcomes in patients with high-risk features that often rely on pathologic nodal status. The impact of SLNB omission on identifying candidates for CDK4/6 inhibitor therapy remains unclear. We evaluated how omission affects eligibility under Choosing Wisely and American Society of Clinical Oncology (ASCO) criteria.

**Methods:**

From the National Cancer Database (2018–2022), we identified women aged ≥ 50 years with hormone-receptor-positive, HER2-negative, clinical T1–2N0M0 breast cancer treated with upfront surgery and SLNB. Omission-eligible cohorts were defined based on Choosing Wisely and ASCO criteria. CDK4/6 inhibitor (abemaciclib and ribociclib) eligibility was assessed using tumor features alone (modeling omission), SLNB findings, and axillary lymph node dissection (ALND) findings. Multivariable logistic regression identified predictors of SLNB determining eligibility.

**Results:**

Among 150,629 patients, 73% met SLNB omission criteria. Positive SLNs were found in 9–10%, and only 2% had pN2–3 disease. SLNB omission would miss 4% (abemaciclib) and 8% (ribociclib) of eligible patients in the Choosing Wisely cohort, and 1 and 8%, respectively, in the ASCO cohort. ALND increased candidacy by only 1%. Approximately 13 SLNBs were required to identify one CDK4/6-eligible patient, and 299 SLNBs were required to identify one patient expected to derive a 5-year invasive disease-free survival benefit.

**Conclusions:**

SLNB omission excludes few patients eligible for adjuvant CDK4/6 inhibitor therapy. These findings support shared decision-making and suggest SLNB should not be performed solely to determine CDK4/6 eligibility.

Sentinel lymph node biopsy (SLNB) has long been the standard approach for axillary staging in early-stage breast cancer, providing prognostic information and guiding adjuvant therapy while avoiding the morbidity associated with axillary lymph node dissection (ALND). Increasing evidence now supports omission of SLNB in select clinical contexts.

The Choosing Wisely (CW) campaign, sponsored by the American Board of Internal Medicine (ABIM) and the Society of Surgical Oncology (SSO), recommends against routine SLNB in women aged ≥ 70 years with hormone-receptor-positive (HR+), HER2-negative (HER2–), clinically node-negative breast cancer, on the basis of studies demonstrating low rates of nodal positivity and axillary recurrence.^1^^,^^2^ More recently, randomized trials including SOUND, INSEMA, and BOOG 13-08 demonstrated noninferiority of SLNB omission in patients with clinically node-negative disease and negative preoperative axillary ultrasound undergoing breast-conserving surgery (BCS).^3–5^ Although these trials had relatively broad inclusion criteria, most participants were postmenopausal and had low-risk disease. These findings informed updated American Society of Clinical Oncology (ASCO) guidelines extending SLNB omission to postmenopausal women aged ≥ 50 years with small, low-grade, clinically node-negative tumors undergoing BCS with negative axillary ultrasound.^6^ As a result, SLNB omission has become an evidence-based option for a substantial and growing proportion of patients.

In parallel, indications for systemic therapy have expanded with the introduction of cyclin-dependent kinase 4/6 (CDK4/6) inhibitors, which improve outcomes in patients with high-risk HR+/HER2– breast cancer. The monarchE trial demonstrated improved invasive disease-free survival (iDFS) and overall survival (OS) with the addition of adjuvant abemaciclib to endocrine therapy, with eligibility largely defined by nodal burden (≥ 4 positive nodes or 1–3 positive nodes with additional high-risk features).^7–9^ The NATALEE trial subsequently demonstrated benefit with ribociclib across a broader population with stage II–III disease, including some patients with node-negative disease.^10^^,^^11^ However, because eligibility for CDK4/6 inhibitor therapy remains closely tied to nodal status, omission of SLNB removes a key determinant of risk and may result in missed opportunities for adjuvant therapy.

These intersecting trends create a clinical tension: surgical de-escalation reduces morbidity^12^ but may limit information used to guide systemic therapy escalation. The frequency and clinical significance of missed CDK4/6 eligibility in this context remain uncertain. Therefore, we aimed to quantify the impact of SLNB omission on CDK4/6 inhibitor eligibility under Choosing Wisely and ASCO criteria, identify patients most likely to be affected, and estimate the clinical consequences of missed eligibility.

## Methods

We performed a retrospective cohort study using the National Cancer Database (NCDB), a hospital-based registry administered by the American College of Surgeons that captures patient-level data from Commission on Cancer-accredited programs and represents approximately 70% of newly diagnosed cancer cases in the USA.^13^^,^^14^ As the NCDB contains de-identified data, this study was exempt from institutional review board oversight.

From the NCDB breast cancer Participant User File, we identified women aged ≥ 50 years diagnosed between 2018 and 2022 with hormone receptor–positive (HR+), HER2-negative, clinical T1–2N0M0 breast cancer who underwent upfront surgery with sentinel lymph node biopsy (SLNB). Patients were excluded for missing or unknown nodal surgery, missing pathologic nodal status, receipt of neoadjuvant systemic therapy, or incomplete tumor receptor data. Although patients receiving neoadjuvant therapy may be eligible for adjuvant CDK4/6 inhibitors, we restricted the cohort to those undergoing upfront surgery to isolate the impact of nodal staging on treatment eligibility.

From this cohort, we defined two subgroups representing patients eligible for SLNB omission on the basis of contemporary guideline-supported criteria, including the Choosing Wisely initiative followed by subsequent ASCO recommendations. The CW cohort included patients aged ≥ 70 years with clinical T1–2 (cT1–2) tumors, without restrictions on tumor grade or breast surgical approach. The ASCO cohort included patients aged 50–69 years with grade 1–2, cT1 tumors undergoing breast-conserving surgery. Preoperative axillary ultrasound is not captured in the NCDB and therefore could not be incorporated into cohort definitions.

We then assessed eligibility for adjuvant CDK4/6 inhibitor therapy using published criteria from the monarchE (abemaciclib) and NATALEE (ribociclib) trials. For monarchE, patients were considered eligible if they had ≥ 4 positive lymph nodes (LNs), or 1–3 positive LNs with at least one additional high-risk feature (tumor size ≥ 5 cm, grade 3, or Ki-67 ≥ 20%). For NATALEE, eligibility included patients with stage IIB–III disease, or stage IIA disease with either ≥ 1 positive LN or grade 2–3 tumors with high genomic risk or Ki-67 ≥ 20%. Genomic risk was defined using 21-gene recurrence score or 70-gene signature testing when available. Because Ki-67 and genomic assay results are incompletely captured in the NCDB, eligibility for CDK4/6 inhibitor therapy may be underestimated in this analysis.

Each patient was evaluated for CDK4/6 inhibitor eligibility under three models: (1) tumor characteristics alone, with stage assigned per American Joint Committee on Cancer (AJCC) 8th edition criteria assuming node-negative disease (to simulate SLNB omission); (2) incorporation of SLNB findings; and (3) incorporation of completion axillary lymph node dissection (ALND) findings among patients who underwent ALND. Incremental changes in eligibility across models were quantified to assess the contribution of nodal staging—and escalation of axillary surgery—to systemic therapy decision-making.

Finally, using observed eligibility differences and published 5-year free iDFS statistics from monarchE and NATALEE,^8^^,^^11^ we estimated the number of SLNB procedures required to (1) identify one additional patient eligible for CDK4/6 inhibitor therapy and (2) yield one additional patient expected to derive invasive disease-free survival benefit.

### Statistical Analysis

Patient, tumor, and treatment characteristics were summarized using descriptive statistics for the overall cohort and each SLNB omission subgroup. The number needed to treat (NNT) was defined as the number of SLNB procedures required to identify one additional patient meeting CDK4/6 inhibitor eligibility criteria. Univariate and multivariable logistic regression models were used to evaluate clinicopathologic predictors of SLNB serving as the determining factor for CDK4/6 inhibitor eligibility within each cohort. A two-sided *p*-value < 0.05 was considered statistically significant. All analyses were performed using SAS version 9.4 (SAS Institute, Cary, NC).

## Results

We identified 150,629 female patients with cT1–2N0M0 HR+/HER2− breast cancer undergoing upfront surgery with SLNB, of whom 56,833 met CW and 52,680 met ASCO SLNB omission criteria (Table 1). In the CW cohort, mean age was 76 ± 4.7 years, 65% had grade 2–3 tumors, 23% had tumors ≥ pT2 at final pathology, 8% had lymphovascular invasion (LVI), 15% had Ki-67 ≥ 20%, and 38% underwent genomic testing, of whom 11% were high risk. In the ASCO cohort, mean age was 61 ± 5.5 years, 55% had grade 2 tumors, 7% were upstaged to ≥ pT2 at final pathology, 5% had LVI, 10% had Ki-67 ≥ 20%, and 57% had a genomic risk score performed, of whom 6% were high risk. Nearly all patients were ER-positive (99.2% and 99.8%, respectively), and most were PR-positive (89% and 92%).

**Table 1:** Clinicopathologic features of females ≥ 50 with cN0 HR+HER2− non-metastatic invasive breast cancer stratified by criteria for SLNB omission

				Choosing Wisely	ASCO
*n*				56,833	52,680
Age (years), mean ± SD				75.72 ± 4.72	61.39 ± 5.49
Race/Ethnicity					
	White			50,060 (88.1)	45,200 (85.8)
	Black			3999 (7.0)	4057 (7.7)
	Asian			1836 (3.2)	2251 (4.3)
	Other/Unknown			938 (1.7)	1172 (2.2)
Charlson–Deyo score					
	0			42,971 (75.6)	43,968 (83.5)
	1			8494 (14.9)	6290 (11.9)
	2			3020 (5.3)	1601 (3.0)
	≥ 3			2348 (4.1)	821 (1.6)
Institution type					
	Community Cancer Center			5133 (9.0)	4131 (7.8)
	Comprehensive Community Cancer Program			26,117 (46.0)	22,178 (42.1)
	Academic/research program			13,362 (23.5)	15,454 (29.3)
	Integrated Network Cancer Program			12,221 (21.5)	10,917 (20.7)
Pathologic tumor size (mm), mean ± SD				16.11 ± 14.47	12.21 ± 13.03
Pathologic tumor stage					
	T0			51 (0.1)	18 (0.0)
	Tis			53 (0.1)	43 (0.1)
	T1			43,474 (76.5)	48,558 (92.2)
	T2			11,956 (21.0)	3854 (7.3)
	T3/4			834 (1.5)	78 (0.1)
	TX			105 (0.2)	58 (0.1)
	Unknown			360 (0.6)	71 (0.1)
Tumor grade					
	1			18,743 (33.0)	23,862 (45.3)
	2			30,725 (54.1)	28,818 (54.7)
	3			6349 (11.2)	
	Unknown			1016 (1.8)	
Estrogen receptor					
	Negative			479 (0.8)	94 (0.2)
	Positive			56,354 (99.2)	52,586 (99.8)
Progesterone receptor					
	Negative			6232 (11.0)	4462 (8.5)
	Positive			50,601 (89.0)	48,218 (91.5)
Ki-67					
	< 20%			20,881 (36.7)	21,173 (40.2)
	≥ 20%			8567 (15.1)	5464 (10.4)
	Unknown			27,385 (48.2)	26,043 (49.4)
Lymphovascular invasion					
	Absent			46,502 (81.8)	45,107 (85.6)
	Present			4756 (8.4)	2913 (5.5)
	Unknown			5575 (9.8)	4660 (8.8)
Genomic score performed					
	Yes			21,498 (37.8)	29,990 (56.9)
		Oncotype			
			≤ 25	16,869 (29.7)	25,429 (48.3)
			> 26	1864 (3.3)	1467 (2.8)
			Unknown	543 (1.0)	687 (1.3)
		Mammaprint			
			Low/intermediate risk	1144 (2.0)	1527 (2.9)
			High risk	480 (0.8)	418 (0.8)
			Unknown	183 (0.3)	194 (0.4)
	No			244 (0.4)	298 (0.6)
	Unknown			35,091 (61.7)	22,392 (42.5)
Breast surgery					
	None/unknown			33 (0.0)	
	Partial mastectomy			44,999 (79.2)	52,680 (100.0)
	Mastectomy			11,801 (20.8)	
Axillary surgery					
	SLNB alone			52,784 (92.9)	49,995 (94.9)
	SNLB then ALND			4049 (7.1)	2685 (5.1)

All values are *n* (%) or mean ± standard deviation unless otherwise indicated

SLNB, sentinel lymph node biopsy; ALND, axillary lymph node dissection

Within the CW cohort, a mean of 2.4 ± 1.7 SLNs were removed; 90% were pN0, while 8% had 1 positive sentinel node (+SLN), 1.5% had 2 +SLNs, and 0.7% had ≥ 3 +SLNs (Table 2). Within the ASCO omission cohort, a mean of 2.5 ± 1.6 SLNs were removed; 91% of patients were pathologically node negative (pN0), while 7% had 1 +SLN, 1% had 2 +SLNs, and 0.4% had ≥ 3 +SLNs.

**Table 2:** Findings at the time of SLNB among females ≥ 50 with cN0 HR+HER2− non-metastatic invasive breast cancer stratified by criteria for SLNB omission

			Choosing Wisely	ASCO
*n*			56,833	52,680
SLNB findings				
	Sentinel nodes removed		2.4 ± 1.7	2.5 ± 1.6
	Sentinel nodes positive			
		0	51,065 (89.9)	48,171 (91.4)
		1	4483 (7.9)	3642 (6.9)
		2	865 (1.5)	644 (1.2)
		≥ 3	420 (0.7)	223 (0.4)
ALND findings (only among 1–2 +SLNs)				
*n*			953	396
	Additional nodes removed		7.7 ± 7.0	5.7 ± 6.4
	Additional nodes positive		1.1 ± 3.3	0.8 ± 2.4
		No	661 (69.4)	297 (75.0)
		Yes	292 (30.6)	99 (25.0)
	Total nodes removed		10.6 ± 7.0	8.3 ± 6.3
	Total nodes positive		2.4 ± 3.3	2.1 ± 2.5
	*Newly meets CDK4/6 criteria*			
		monarchE	64 (6.7)	39 (9.8)
		NATALEE	0 (0.0)	0 (0.0)
ALND findings (only among ≥ 3 +SLNs)				
n			181	106
	Additional nodes removed		9.5 ± 7.4	11.0 ± 8.0
	Additional nodes positive		3.3 ± 4.2	2.8 ± 4.8
		No	75 (41.4)	52 (49.1)
		Yes	106 (58.6)	54 (50.9)
	Total nodes removed		15.0 ± 7.0	16.9 ± 7.6
	Total nodes positive		7.2 ± 4.4	6.6 ± 5.4
	*Newly meets CDK4/6 criteria*			
		monarchE	32 (17.7)	21 (19.8)
		NATALEE	0 (0.0)	0 (0.0)

All values are *n* (%) or mean ± standard deviation unless otherwise indicated

SLNB, sentinel lymph node biopsy; ALND, axillary lymph node dissection; SLNs, sentinel lymph nodes

### Missed CDK4/6 Candidacy

**FIG. 1:**
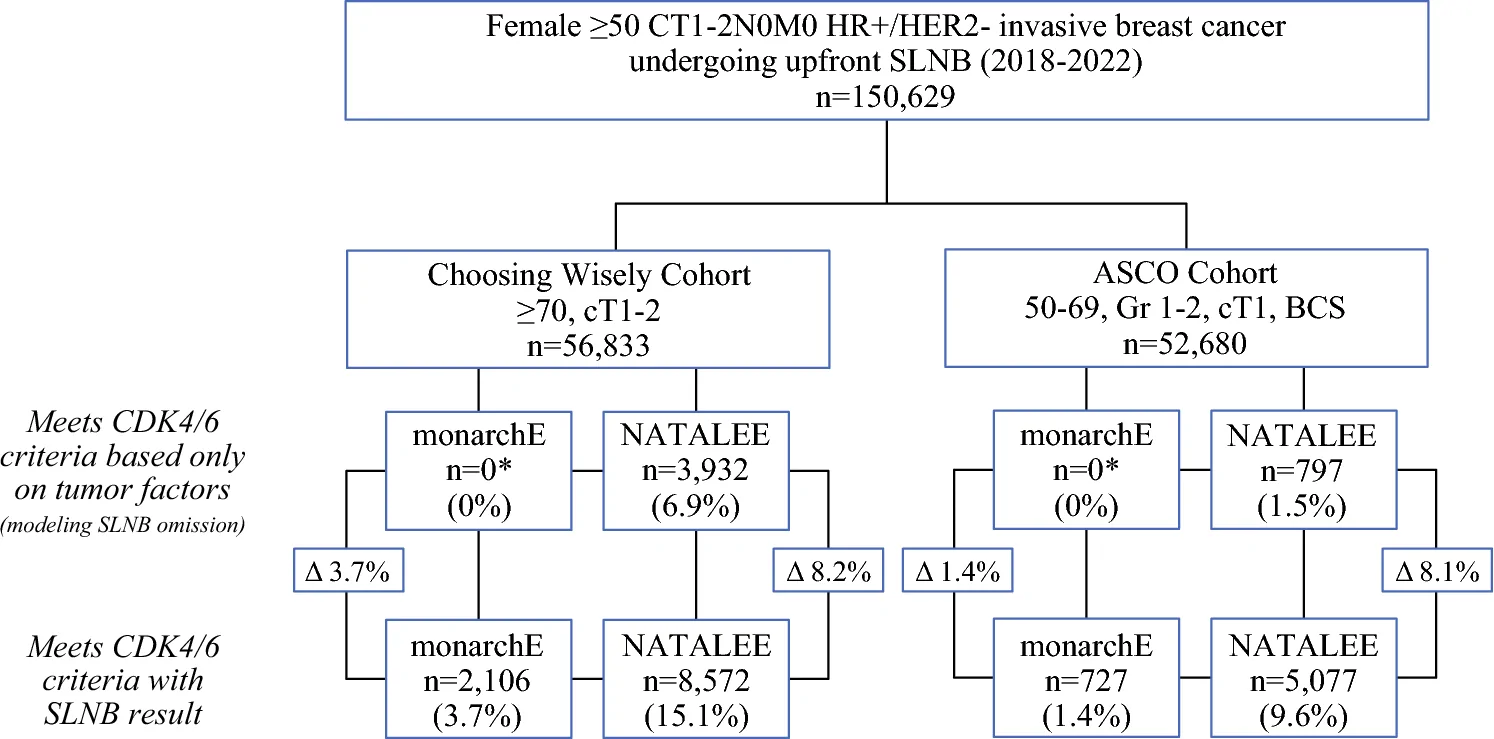
Impact of sentinel lymph node biopsy (SLNB) on CDK4/6 inhibitor eligibility under Choosing Wisely and ASCO omission criteria. Incremental eligibility from SLNB is defined as the difference between tumor-based eligibility (modeling omission) and eligibility after SLNB (Δ). * Abemaciclib (monarchE) eligibility requires nodal positivity; therefore, no patients met criteria based on tumor features alone

When considering tumor characteristics alone (assuming node-negative disease), no patients met monarchE criteria for abemaciclib, as nodal positivity is required for eligibility (Fig. 1). Regarding ribociclib candidacy, 6.9% of the CW cohort and 1.5% of the ASCO cohort met eligibility criteria per NATALEE without the information obtained from SLNB.

After incorporating SLNB findings, 3.7% of CW patients and 1.4% of ASCO patients met criteria for adjuvant abemaciclib, while 15.1 and 9.6%, respectively, met criteria for adjuvant ribociclib. Accordingly, omission of SLNB would result in missed eligibility for abemaciclib in 3.7% of CW and 1.5% of ASCO patients, and for ribociclib in 8.2 and 8.1%, respectively.

We performed univariate and multivariable logistic regression to identify clinicopathologic factors associated with SLNB serving as the determining factor for CDK4/6 eligibility. In the ASCO cohort (Table 3), grade 2 tumors (versus grade 1; OR 0.20, *p* < 0.001) and Ki-67 ≥20% (OR 0.05, *p* < 0.001) were associated with a lower likelihood that SLNB determined CDK4/6 eligibility, whereas lymphovascular invasion (OR 1.60, *p* < 0.001) and PR positivity (OR 1.70, *p* = 0.03) were associated with a higher likelihood. Similar associations were observed in the CW cohort (Table 4); however, higher clinical tumor stage was additionally associated with a lower likelihood that SLNB determined CDK4/6 eligibility.

**Table 3:** Factors associated with missed CDK4/6 eligibility if SLNB were omitted in the SOUND/INSEMA cohort

		Univariate			Multivariable		
		OR	95% CI	*p*	OR	95% CI	*p*
Age (years)		0.99	0.98–1.00	0.15	0.98	0.95–1.00	0.09
Race/ethnicity							
	White (ref)	–	–	–	–	–	–
	Black	0.83	0.65–1.07	0.14	1.05	0.69–1.58	0.83
	Asian	0.77	0.54–1.09	0.14	0.72	0.40–1.32	0.29
Charlson–Deyo score							
	0 (ref)	–	–	–	–	–	–
	1	1.08	0.85–1.36	0.53	1.28	0.86–1.92	0.23
	2	1.13	0.73–1.77	0.58	0.79	0.36–1.73	0.56
	≥ 3	1.51	0.81–2.81	0.19	1.29	0.51–3.29	0.59
Insurance status							
	Not insured (ref)	–	–	–	–	–	–
	Private insurance	1.08	0.58–2.01	0.80	1.45	0.49–4.26	0.50
	Medicaid	1.10	0.57–2.15	0.77	1.16	0.36–3.71	0.80
	Medicare	1.04	0.55–1.94	0.91	1.47	0.48–4.49	0.50
	Other government	0.64	0.28–1.51	0.31	1.11	0.27–4.64	0.88
No high school degree							
	≥ 17.6% (ref)	–	–	–	–	–	–
	10.9–17.5%	1.06	0.83–1.36	0.64	1.17	0.78–1.76	0.44
	6.3–10.8%	1.01	0.79–1.28	0.97	0.97	0.63–1.48	0.88
	< 6.3%	1.24	0.96–1.61	0.11	1.18	0.72–1.93	0.52
Median income							
	< $40,227 (ref)	–	–	–	–	–	–
	$40,227–50,353	1.03	0.78–1.37	0.83	0.85	0.54–1.36	0.50
	$50,354–63,332	0.98	0.75–1.30	0.91	0.96	0.60–1.53	0.86
	≥ $63,333	1.08	0.83–1.39	0.57	1.13	0.70–1.83	0.62
Institution type							
	Community Cancer Center (ref)	–	–	–	–	–	–
	Comprehensive Community Cancer Program	0.73	0.55–0.99	0.04	0.83	0.52–1.34	0.45
	Academic/research program	0.88	0.65–1.20	0.43	0.87	0.53–1.44	0.59
	Integrated Network Cancer Program	0.85	0.62–1.18	0.34	0.90	0.53–1.52	0.69
Tumor grade							
	1 (ref)	–	–	–	–	–	–
	2	0.14	0.11–0.19	< 0.001	0.20	0.12–0.33	< 0.001
Ki-67							
	< 20% (ref)	–	–	–	–	–	–
	≥ 20%	0.04	0.03–0.05	< 0.001	0.05	0.04–0.07	< 0.001
Lymphovascular invasion positive		1.16	0.97–1.39	0.10	1.69	1.27–2.26	< 0.001
Estrogen receptor positive		1.80	0.40–8.15	0.45	0.35	0.04–3.09	0.34
Progesterone receptor positive		2.03	1.59–2.58	< 0.001	1.70	1.05–2.74	0.03

OR, odds ratio; CI, confidence interval

**Table 4:** Factors associated with missed CDK4/6 eligibility if SLNB were omitted in the Choosing Wisely cohort

		Univariate			Multivariable		
		OR	95% CI	*p*	OR	95% CI	*p*
Age (years)		0.98	0.97–0.98	< 0.001	0.98	0.97–1.00	0.09
Race/ethnicity							
	White (ref)	–	–	–	–	–	–
	Black	0.86	0.73–1.00	0.05	1.06	0.75–1.50	0.73
	Asian	0.74	0.59–0.93	0.01	0.99	0.61–1.62	0.98
Charlson–Deyo score							
	0 (ref)	–	–	–	–	–	–
	1	0.96	0.85–1.08	0.48	0.99	0.77–1.28	0.95
	2	0.85	0.71–1.01	0.07	0.71	0.47–1.08	0.11
	≥ 3	1.02	0.83–1.24	0.87	1.20	0.76–1.88	0.44
Insurance status							
	Not insured (ref)	–	–	–	–	–	–
	Private insurance	1.18	0.59–2.37	0.65	0.39	0.10–1.59	0.19
	Medicaid	1.11	0.51–2.41	0.80	0.53	0.11–2.53	0.43
	Medicare	1.12	0.56–2.21	0.76	0.45	0.11–1.75	0.25
	Other government	1.45	0.60–3.51	0.41	0.47	0.08–2.98	0.42
No high school degree							
	≥ 17.6% (ref)	–	–	–	–	–	–
	10.9–17.5%	1.01	0.88–1.17	0.84	1.10	0.82–1.46	0.54
	6.3–10.8%	1.06	0.92–1.21	0.41	1.12	0.82–1.52	0.48
	< 6.3%	0.98	0.85–1.14	0.81	0.99	0.70–1.40	0.94
Median income							
	< $40,227 (ref)	–	–	–	–	–	–
	$40,227–50,353	1.16	1.00–1.35	0.06	1.36	0.99–1.87	0.06
	$50,354–63,332	0.97	0.84–1.13	0.68	1.12	0.81–1.55	0.49
	≥ $63,333	1.04	0.90–1.19	0.61	1.02	0.73–1.44	0.89
Institution type							
	Community Cancer Center (ref)	–	–	–	–	–	–
	Comprehensive Community Cancer Program	1.02	0.88–1.18	0.80	1.17	0.86–1.60	0.33
	Academic/research program	1.06	0.90–1.25	0.49	0.88	0.62–1.24	0.46
	Integrated Network Cancer Program	1.03	0.88–1.22	0.69	1.01	0.71–1.44	0.95
Clinical tumor stage							
	T1 (ref)	–	–	–	–	–	–
	T2	0.24	0.22–0.26	< 0.001	0.19	0.16–0.24	< 0.001
Tumor grade							
	1 (ref)	–	–	–	–	–	–
	2	0.22	0.19–0.26	< 0.001	0.38	0.27–0.54	< 0.001
	3	0.07	0.06–0.08	< 0.001	0.25	0.17–0.37	< 0.001
Ki-67							
	< 20% (ref)	–	–	–	–	–	–
	≥ 20%	0.04	0.04–0.05	< 0.001	0.05	0.04–0.06	< 0.001
Lymphovascular invasion positive		1.49	1.35–1.64	< 0.001	2.10	1.72–2.57	< 0.001
Estrogen receptor positive		4.56	2.79–7.45	< 0.001	1.38	0.56–3.41	0.49
Progesterone receptor positive		2.22	1.96–2.52	< 0.001	2.55	1.91–3.40	< 0.001

OR, odds ratio; CI, confidence interval

### Number Needed to Treat

We next quantified the number of SLNB procedures required to identify one patient eligible for CDK4/6 inhibitor therapy. An estimated 27 CW patients and 73 ASCO patients would need to undergo SLNB to identify one patient eligible for abemaciclib, whereas 13 patients in each cohort would be required to identify one patient eligible for ribociclib (Table 5).

**Table 5:** Number of sentinel lymph node biopsies required to identify one CDK4/6-eligible patient and number needed to treat to achieve one 5-year invasive disease-free survival benefit

SLNBs required to identify one CDK4/6-eligible patient		
	Choosing Wisely	ASCO
Abemaciclib	27	73
Ribociclib	13	13
SLNBs required to identify one patient with 5-year iDFS benefit attributable to CDK4/6 use		
	Choosing Wisely	ASCO
Abemaciclib	378	1022
Ribociclib	299	299

SLNB, sentinel lymph node biopsy; iDFS, invasive disease-free survival

Using results from the monarchE and NATALEE trials, we estimated that the number needed to treat (NNT) to achieve a 5-year iDFS benefit was 14 and 23, respectively. We then estimated the number of SLNB procedures required to identify one patient expected to derive a 5-year iDFS benefit from CDK4/6 inhibitor therapy (Table 5). An estimated 378 CW patients and 1022 ASCO patients would need to undergo SLNB for one patient to derive iDFS benefit from abemaciclib, whereas 299 SLNB procedures would be required in each cohort for one patient to benefit from ribociclib.

Therefore, assuming a 5% risk of lymphedema following SLNB, approximately one patient would develop lymphedema for every patient identified as eligible for CDK4/6 inhibitor therapy if SLNB were routinely performed in CW and ASCO populations. Importantly, an estimated 15 patients would develop lymphedema for every one patient expected to derive a 5-year iDFS benefit from CDK4/6 therapy.

### Axillary Lymph Node Dissection

Among the combined CW and ASCO cohorts, 9634 patients (9%) had 1–2 +SLNs and 643 (0.6%) had ≥ 3 +SLNs. ALND was performed in 14 and 44% of these groups, respectively (Table 2). Among patients with 1–2 +SLNs, for whom ALND is not routinely recommended, 10% of CW patients and 7% of ASCO patients became eligible for abemaciclib on the basis of additional nodal disease identified at ALND, while no patients became eligible for ribociclib. Among patients with ≥ 3 +SLNs, 18% of CW and 20% of ASCO patients became eligible for abemaciclib on the basis of pathologic findings after ALND, and no additional patients became eligible for ribociclib. Taken together, ALND increased CDK4/6 inhibitor eligibility in only 1% of patients in the combined CW and ASCO cohorts.

## Discussion

In this large, contemporary national cohort of women aged 50 years or older with HR+/HER2− early breast cancer, we found that omission of SLNB would exclude only a small proportion of patients from meeting criteria for adjuvant CDK4/6 inhibitor therapy. Overall, a minority of omission-eligible patients qualified for CDK4/6 inhibitors even when SLNB was performed, with eligibility rates varying by cohort and agent, and SLNB identified only a small absolute increase in additional eligible individuals.

The impact of SLNB on CDK4/6 inhibitor eligibility depends on the interplay between tumor biology and nodal staging. In our analysis, tumor features that independently confer CDK4/6 eligibility, such as higher grade or elevated Ki-67, reduced the incremental value of nodal staging, as these patients would qualify for therapy regardless of SLNB findings. In contrast, features such as lymphovascular invasion and progesterone receptor status, which are associated with nodal positivity but are not themselves eligibility criteria, increased the likelihood that SLNB findings would determine candidacy. Notably, tumor size did not meaningfully influence SLNB-driven eligibility, likely because most tumors remained within T1–2 categories and upstaging to ≥ pT3, which would independently confer eligibility, was uncommon. Together, these findings suggest that the clinical value of SLNB is greatest in patients with intermediate-risk features, where nodal information has the highest potential to alter systemic therapy recommendations.

Our findings extend and clarify prior work. Two recent single-institution studies have suggested that omission of SLNB in patients meeting ASCO criteria has minimal impact on identifying candidates for adjuvant CDK4/6 inhibition. In a retrospective analysis of 948 SLNB omission-eligible patients treated at Tübingen University Hospital, Kandzi et al. found that, while 15% of patients had occult nodal disease identified at SLNB, only 2% of the overall cohort met criteria for abemaciclib and 11% met criteria for ribociclib.^15^ They further estimated that 291 SLNB procedures would be required to prevent one recurrence with CDK4/6 inhibitor therapy, closely mirroring our findings. Similarly, Heidinger et al. reported outcomes from 309 omission-eligible patients treated at the University Hospital of Basel, with a 15% node-positive rate and 13–30 SLNB procedures required to identify one CDK4/6-eligible patient and at least 350 SLNBs required to prevent one iDFS event.^16^ In a complementary analysis of the NCDB (2018–2020), Wanis et al. evaluated the impact of SLNB omission on adjuvant decision-making and found that, among 103,528 women aged ≥ 50 years with cT1N0 HR+/HER2− breast cancer, nodal pathology increased CDK4/6 inhibitor eligibility by only 2% for abemaciclib and 8% for ribociclib.^17^ Taken together, these studies consistently demonstrate that SLNB omission does not result in a substantial number of missed candidates for adjuvant CDK4/6 inhibition. Our analysis builds upon this foundation by evaluating a larger, nationally representative cohort and by directly comparing outcomes across different SLNB omission guidelines. Because omission criteria differ between Choosing Wisely and ASCO recommendations, the underlying nodal disease burden—and therefore the likelihood of meeting CDK4/6 eligibility thresholds—varies between these groups. As such, our findings provide clinically relevant data to inform shared decision-making in patients aged 50–69 years compared with those aged ≥ 70 years.

The clinical implications of these findings are substantial. As surgical de-escalation strategies expand, SLNB has come under increasing scrutiny owing to its associated morbidity, including lymphedema, sensory changes, shoulder dysfunction, and procedural costs. However, de-escalation must be pursued in an oncologically safe manner to avoid missing opportunities for systemic therapy escalation. In this context, our results demonstrate that, for the vast majority of patients meeting guideline-based criteria for omission, SLNB does not meaningfully alter eligibility for CDK4/6 inhibitor therapy. Even when SLNB does change eligibility, the absolute number of patients affected is small, and the downstream clinical benefit is likely limited, although this may vary by agent; adjuvant abemaciclib has demonstrated an overall survival benefit in high-risk populations, whereas long-term survival data for ribociclib remain immature. Our analysis shows that hundreds of SLNB procedures are required to identify a single patient who would derive an iDFS benefit from CDK4/6 therapy, while a comparable number of patients would be exposed to the risk of lymphedema. Thus, the marginal oncologic benefit gained from routine SLNB in this population is low and must be weighed against the well-established morbidity of axillary surgery. These data support SLNB omission in appropriately selected patients and reinforce a treatment paradigm that increasingly relies on tumor biology rather than nodal status to guide systemic therapy decisions. Notably, a subset of patients in the Choosing Wisely cohort underwent mastectomy, for whom omission of SLNB is less well established and may be less consistently adopted in clinical practice, which may influence the generalizability of these findings.

Importantly, our findings also reinforce the central role of shared decision-making. Although eligibility for CDK4/6 inhibitor therapy under trial criteria is binary, the magnitude of clinical benefit is not uniform. Older adults (who form the entirety of the CW cohort) are underrepresented in pivotal trials and may derive less absolute benefit, experience greater toxicity, and face higher competing mortality risks. Conversely, some younger or medically fit patients may reasonably elect to undergo SLNB to ensure that all potential systemic therapy options remain available, particularly if they prioritize maximal oncologic treatment. In addition, multidisciplinary teams may, in select cases, favor surgical nodal staging to guide treatment recommendations. Institutional protocols that support multidisciplinary evaluation of clinicopathologic features both before and after surgery are critical to safely implementing de-escalation strategies, and any changes in tumor characteristics on surgical pathology (such as increased grade or size) should prompt re-evaluation of the need for SLNB.

Finally, our findings further underscore the limited role of ALND in determining CDK4/6 inhibitor eligibility. We have previously demonstrated in an NCDB analysis that ALND rarely identifies sufficient additional nodal disease to alter systemic therapy recommendations in patients who are not eligible for CDK4/6 inhibitors after SLNB, findings that have been corroborated by analyses of the SENOMAC trial and other cohorts.^18^^,^^19^ In the present study, ALND provided minimal incremental information for determining CDK4/6 inhibitor eligibility while substantially increasing the risk of morbidity, reinforcing that ALND should not be performed solely to determine CDK4/6 candidacy in patients with limited nodal disease.

This study has limitations inherent to retrospective registry analyses. The NCDB lacks data on recurrence, treatment toxicity, and adherence, limiting evaluation of downstream clinical outcomes. Nodal imaging data, including axillary ultrasound, are not captured, likely resulting in conservative overestimation of missed eligibility. Genomic testing results and Ki-67 were available for approximately half of patients, which may underestimate CDK4/6 eligibility. Trial-based criteria were used to model CDK4/6 eligibility; however, recent Food and Drug Administration (FDA) updates to abemaciclib criteria were not incorporated, and real-world prescribing may be more individualized. Despite these limitations, the study’s large sample size, contemporary timeframe, and stratification by guideline-defined omission criteria provide a robust and clinically meaningful estimation of the impact of SLNB omission.

## Conclusions

SLNB omission under ASCO and Choosing Wisely criteria excludes a small minority of patients who would otherwise meet eligibility for adjuvant CDK4/6 inhibitors. The limited incremental oncologic yield of SLNB, combined with its morbidity, supports guideline-based omission and discourages routine use of SLNB solely to determine CDK4/6 candidacy. These findings emphasize the importance of individualized, multidisciplinary decision-making and highlight that the central considerations surrounding SLNB omission extend beyond staging to thoughtfully balancing surgical harm against the realistic incremental benefit of systemic therapy in contemporary breast cancer care.
